# Model-Based Deconvolution of Cell Cycle Time-Series Data Reveals Gene Expression Details at High Resolution

**DOI:** 10.1371/journal.pcbi.1000460

**Published:** 2009-08-14

**Authors:** Dan Siegal-Gaskins, Joshua N. Ash, Sean Crosson

**Affiliations:** 1 Mathematical Biosciences Institute, Ohio State University, Columbus, Ohio, United States of America; 2 Department of Plant Cellular and Molecular Biology, Ohio State University, Columbus, Ohio, United States of America; 3 Plant Biotechnology Center, Ohio State University, Columbus, Ohio, United States of America; 4 Department of Electrical and Computer Engineering, Ohio State University, Columbus, Ohio, United States of America; 5 Department of Biochemistry and Molecular Biology, University of Chicago, Chicago, Illinois, United States of America; 6 The Committee on Microbiology, University of Chicago, Chicago, Illinois, United States of America; Johns Hopkins University, United States of America

## Abstract

In both prokaryotic and eukaryotic cells, gene expression is regulated across the cell cycle to ensure “just-in-time” assembly of select cellular structures and molecular machines. However, present in all time-series gene expression measurements is variability that arises from both systematic error in the cell synchrony process and variance in the timing of cell division at the level of the single cell. Thus, gene or protein expression data collected from a population of synchronized cells is an inaccurate measure of what occurs in the average single-cell across a cell cycle. Here, we present a general computational method to extract “single-cell”-like information from population-level time-series expression data. This method removes the effects of 1) variance in growth rate and 2) variance in the physiological and developmental state of the cell. Moreover, this method represents an advance in the deconvolution of molecular expression data in its flexibility, minimal assumptions, and the use of a cross-validation analysis to determine the appropriate level of regularization. Applying our deconvolution algorithm to cell cycle gene expression data from the dimorphic bacterium *Caulobacter crescentus*, we recovered critical features of cell cycle regulation in essential genes, including *ctrA* and *ftsZ*, that were obscured in population-based measurements. In doing so, we highlight the problem with using population data alone to decipher cellular regulatory mechanisms and demonstrate how our deconvolution algorithm can be applied to produce a more realistic picture of temporal regulation in a cell.

## Author Summary

Time-series analyses of cellular regulatory processes have successfully drawn attention to the importance of temporal regulation in biological systems. A number of model systems can be synchronized such that data collected on cell populations better reflect the dynamic properties of the individual cell. However, experimental synchronization is never perfect, and the degree of synchrony that does exist at the outset of an experiment is quickly lost over time as cells grow at different rates and enter different developmental or physiological states on cell division. Thus, data collected from a population of synchronized cells can lead to incorrect models of temporal regulation. Here we demonstrate that the problem of relating population data to the individual cell can be resolved with a computational method that effectively removes the effects of both imperfect synchrony and time-dependent loss of synchrony. Application of this deconvolution algorithm to a cell cycle time-series data set from the model bacterium *Caulobacter crescentus* uncovers critical temporal details in the expression of essential genes that are not evident in the raw population-based data. The deconvolution routine presented here is a robust and general tool for extracting biochemical parameters of the average single cell from population time-series data.

## Introduction

Recent technological advances have made feasible studies of biological systems at the single-cell level [1]–[4]. However, our current understanding of single-cell biochemistry and physiology has been largely inferred from averaged population measurements that often mask individual cell dynamics and lead to a distorted picture of cell behavior. Such cell population data can be difficult to reconcile with single-cell models, such as those that attempt to describe cell-cycle-dependent gene expression kinetics [5]–[7]. In particular, mathematical models of single cells that rely on population data for constraints on biochemical parameters may arrive at incorrect conclusions.

Among the properties hidden by population averaging is cell-to-cell variability, such as that found in gene expression and protein production [8]–[11]. We refer to the natural variation found between cells at the same position in their cell cycles as synchronous variability. A population experiment in which synchronous variability is the only source of variability can at most yield the average of the observable of interest (e.g., gene expression levels). However, in addition to the inherent synchronous variability, typical time-series experiments on cells contain a significant asynchronous variability: even if cells have been physically or chemically synchronized, individual cells within a synchronized population exist at variable points in their respective cell cycles. As a result, the extraction of ‘true’ temporal data from such populations is difficult, since contributions from cells in different stages of the cell cycle are averaged.

From a mathematical perspective, population asynchrony may be modeled as a kernel function that maps the average of an observable in the absence of asynchronous variability to the value measured at the population level. Population asynchrony has been modeled in yeast as both a time-dependent [12],[13] and time-independent [14] source of variability. With an accurate asynchrony model, extracting the average of an observable becomes an inverse problem for which established regularization methods can be used. These computational methods can effectively remove from population data artifacts that are due solely to asynchrony, or uncover features that are masked by population averaging [13]–[15]. The resulting data is thus better suited for comparison with single-cell models and parameter estimation.

**Figure 1:**
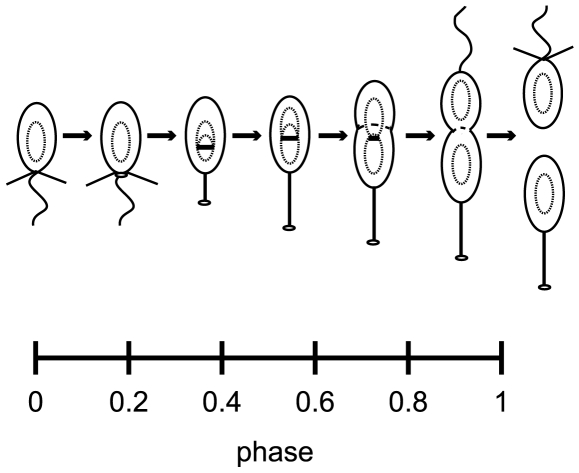
*Caulobacter* cell cycle shown with phase axis. *Caulobacter* begins its cycle as a motile ‘swarmer’ (SW) cell and differentiates to a non-motile ‘stalked’ (ST) state. Division produces two morphologically distinct cells. The cell-cycle phase concept is described in the Model section.

Population asynchrony characterization is most easily done with a synchronizable system such as the dimorphic bacterium *Caulobacter crescentus*. *Caulobacter* begins its cycle as a motile ‘swarmer’ (SW) cell and differentiates to a non-motile ‘stalked’ (ST) cell just prior to the initiation of DNA replication. The SW stage is thus analogous to the G1 phase of the eukaryotic cell cycle, and the ST stage is analogous to the S and G2 phases [16]. At the SW-to-ST transition, the flagellum is released and a narrow cylindrical extension of the cell envelope (the ‘stalk’) is grown in its place. A new flagellar assembly is constructed at the pole opposite the stalk as the cell cycle progresses, and on cell division, a new motile, chemotactic SW cell is spawned. The remaining ST cell immediately commences another round of DNA replication and division while the SW cell begins the full cell cycle (Fig. 1). Centrifugation of a mixed culture of *Caulobacter* in Ludox or Percoll separates SW cells from all other cell types, so that nearly pure cultures of SW cells can be easily obtained [17],[18]. However, even a perfectly pure culture of SW cells includes a mixture of new and old SW cells, and variance in the cell cycle times of individual cells within this synchronized population leads to a further increase in the heterogeneity of the population as time-series experiments progress. Additional heterogeneity is introduced following cell division, as each dividing cell results in both a SW and ST cell. Thus, even a perfectly synchronized population develops a significant and time-dependent population asynchrony.

We propose a simple model for the time-dependent distribution of *Caulobacter* cell types in a population during synchronized growth. Our model accurately matches observed distributions of synchronized *Caulobacter* cells during a time-series experiment, and may be extended to any organism for which the synchrony state can be characterized—particularly those that undergo asymmetric division. We then combine a generalization of deconvolution with our *Caulobacter* distribution model to extract the “single-cell”-like synchronous average of gene expression profiles from published cell cycle microarray data. The resulting expression profiles more accurately predict the cell-cycle position and size of gene expression peaks, display new features not evident in the original microarray data set, and demonstrate robustness to uncertainty in model parameters. This represents a new advance in the study of cell-cycle dependent gene expression in *Caulobacter*. The deconvolution method presented herein can be generally applied to characterize time-dependent processes in a variety of biological model systems.

## Model

### Cell-type distribution model

To effectively remove the effects of population asynchrony from measured data, we must first establish a model describing the temporal position of cells within their own cell cycles and how they are distributed in the population. In this section we develop this model in the context of *Caulobacter*, however, the modeling framework and deconvolution procedure remain generally applicable to other model systems.

We refer to the position of a cell within its own cell cycle as the cell's *phase* 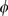, and define it to be a number between zero and one. By our definition 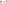 represents a new SW cell and 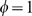 is a predivisional cell at the instant before cell division (Fig. 1). In addition to 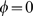 and 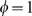, other phases of interest are the phase at which the cell transitions from SW to ST, from ST to early predivisional cell (EPD), and from early predivisional to late predivision cell (LPD). The concept of a cell cycle phase has been used previously, referred to as either the cell division unit or cell cycle unit [19]–[21].

At time *t* following synchronization, we assume that each cell of a large population of 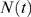 cells is described by three variables:

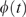
: the phase of the cell at time *t*

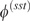
: the SW-to-ST transition phase

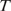
: the total cycle time (minutes)

All three of these cell-specific quantities are random variables; 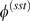 and 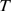 do not change with time, and 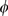 is time dependent. Therefore, a probability density function (PDF) may be written to describe the distribution of these parameters in a population of cells at a given time *t*

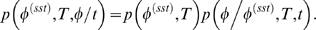
(1)The variables 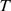 and 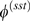 are assumed to be independent and normally-distributed (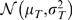 and 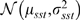). The *Caulobacter* cell cycle time coefficient of variation (COV) was previously determined to be 0.13 [1], i.e. 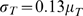. We assume that 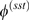 has the same COV and a mean value of 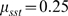, consistent with previous reports [17],[22]. For notational simplicity, we let 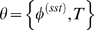 and rewrite the Eq. (1) as 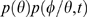, with 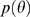 given as the products of the two independent normal distributions just described.

The conditional distribution 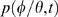 is based on a phase evolution model that is firmly rooted in experimental observations. We begin by considering a single cell (indexed *k*) described by the variables 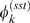 and 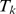. This cell progresses through the phases of its own cell cycle with a ‘velocity’ of 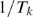 as experiment time passes; that is, 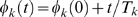 for 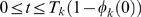. When 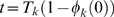, and the cell reaches the end of its cycle, two daughter cells emerge at different cell cycle phases: the new SW (characterized by 
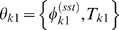
) cell begins at 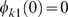 and the new ST cell (now characterized by 
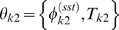
) begins at the SW-to-ST transition phase 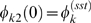. The new SW-to-ST transition phases and cell cycle times, 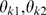, are redrawn from their respective distributions.

### Mapping phase-varying gene expression in single cells to measurements at the population-level

Having constructed a model for the distribution of cell types, we now show how this distribution can be used to map gene expression at the single-cell level to the expression data derived from cellular populations. The signal intensity measured in a typical microarray experiment is proportional to the population-level concentration of the measured species [23]. Thus, for each gene *j* in an RNA expression assay, the signal intensity 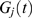 at measurement time *t* is
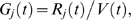
(2)where 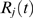 is the number of RNA transcripts in the population and 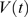 is the total cellular volume. For a large number of cells 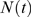, the total population volume is
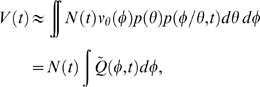
(3)where 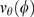 is the volume of a cell with 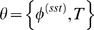 at phase 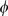, and 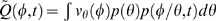 is the expectation of a single cell's volume over 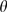. Similarly, the total number of RNA transcripts at time *t* for a given gene *j* is
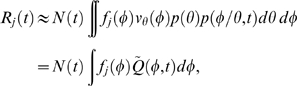
(4)where 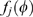 is the synchronous average cycle-dependent expression of gene *j*, i.e., the average expression of all cells at the exact same phase. The expression level 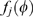 has units (# transcripts/volume). Note that we may substitute the synchronous average expression function for the true single-cell function in the above equation because the synchronous cell-to-cell variability is independent of 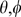 (see supplementary Text S1 for more details).

It has been previously shown that the *Caulobacter* division plane is not located at the center of the cell, rather the cell volume is partitioned 40% SW cell to 60% ST cell [24]. We use this fact to construct a simple piecewise linear approximation for the volume 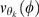 of cell *k*, with parameters 
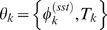
, as a function of cell cycle phase
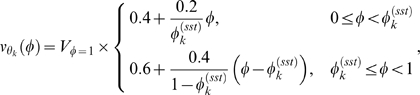
(5)where 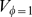 is the cell volume at 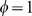 just prior to division. We have assumed that the variance of the final cell size distribution is small so that 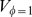 is effectively constant across all cells.

Using the above approximations, the total concentration of gene *j* transcripts at time *t* (Eq. (2)) can then be written as an integral transform
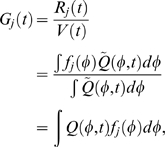
(6)where 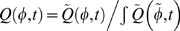 is the kernel of the transform, and has the intrepretation of a fractional volume density. That is, 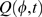 represents the fraction of the total population volume at time *t* that exists in (a small interval around) phase 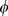.

### Evaluation of 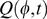


The kernel mapping function 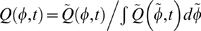 depends on 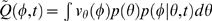, where the volume 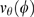 and probability 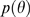 are known functions. However, the functional form of 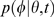 is complicated by the facts that cells evolve at different rates and that new cells are being generated at different phases. We therefore resort to simulation methods in order to evaluate 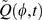 and 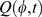.

**Figure 2:**
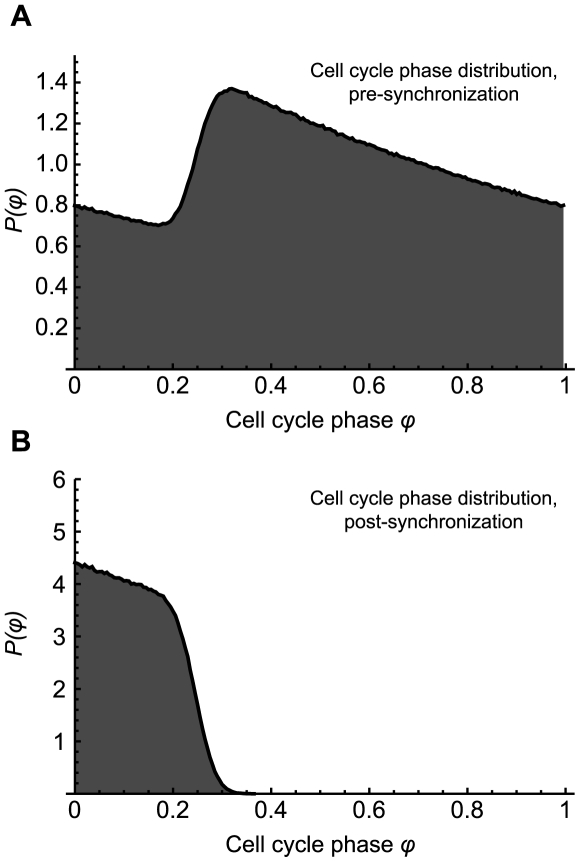
*Caulobacter* cell cycle phase distribution, before and after synchronization. (A) The simulated steady state cell cycle phase distribution shown here is achieved after ∼10 average cell division times. Each cell *k* in the population progresses through the phases of its own cell cycle with a ‘velocity’ of 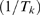 as time passes, and when the cell reaches the end of its cycle, a new SW cell and new ST cell emerge. The steady state is independent of any initial phase distribution. (B) From the steady state distribution the simulated cells are synchronized as real cells are: by keeping only those cells in the SW stage and rejecting all ST cells.

The rule-based *Caulobacter* cell-type phase evolution model described above enables us to simulate cell populations and growth. An initial population of cells was subjected to simulated growth for a length of time equal to 10 average cell division times. We observe, empirically, that this amount of time is sufficient in order to obtain a steady state population of cells whose phase distribution is independent of the initial seed population. The synchronized population is then drawn from the steady state population by keeping only those cells in the SW state and rejecting all others. The steady state distribution is shown in Fig. 2A, and the distribution of synchronized cells is shown in Fig. 2B. After synchronization, time 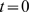 is declared, and the expression experiment begins. Our results utilized 10^6^ synchronized cells at 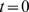.

Rewriting 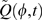 as
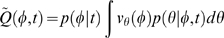
(7)we see that 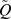 is the product of i) the probability (density) of observing 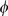 at time *t* and ii) the average cell volume at time *t* conditioned on phase 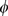. These two quantities are evaluated through the simulation by allowing the synchronized cells to evolve until a desired time *t* is reached and the current population of cells, 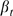, can be used to evaluate 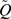.

For a desired 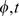, let the 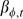 denote the indices of the cells with phases approximately equal to 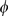


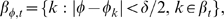
(8)where 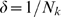 is a small interval. The marginal probability density is approximated as
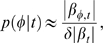
(9)with 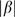 denoting the cardinality of set 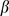. The expected volume is similarly calculated as
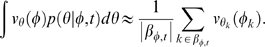
(10)he integral 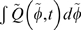 may be approximated using quadrature methods on a sampled version of 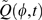 or by observing that the integral is the expected volume over all cells at time *t*, which is calculated by substituting 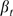 for 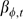 in the right hand side of Eq. (10).

**Figure 3:**
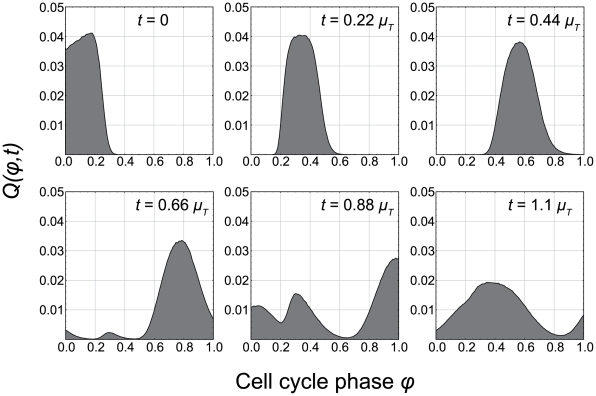
The integral transform kernel 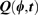 describes the time-dependent population asynchrony. At the outset of the experiment, all cells can be found in the SW stage. The distribution broadens as experiment time goes on and cells progress through their cycles at different rates. Following division, new peaks emerge in the distribution as daughter cells enter the population with different cell cycle phases: SW cells with 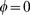 and new ST cells with 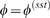. We observe that the kernel structure is highly time dependent and not well-modeled by any common form, such as a Gaussian. Experiment time is shown relative to the average cell cycle time 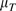.

Hence, Eq. (9) and Eq. (10), combined with a rule-based model of the evolution of cell types within a population enable us to compute the kernel transformation needed to invert population measurements into single-cell data. The kernel 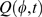 is shown for six different times following synchronization in Fig. 3. The time evolution of 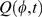 is also shown with 0.5 minute resolution in supplementary Video S1. We observe that the kernel structure is highly time dependent and not well-modeled by any common form. As such, any attempts to reconstruct expression functions by deconvolving with fixed kernels, e.g. a Gaussian kernel, will lead to poor results.

### Estimating synchronous average single-cell gene expression using cubic splines

With the complete noiseless measurement model given as the integral equation in Eq. (6), extracting average single-cell information involves solving the integral equation for 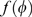 given a set of concentration measurements 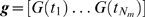 (the *j* subscripts on 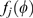 and 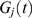 are dropped for notational clarity). Because the number of measurements 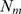 is finite and small, the inversion process is ill-posed and requires a degree of regularization, i.e., the introduction of additional information. Since 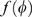 is a physical process, we expect it to be a smooth continuous function and model it as a natural cubic spline. That is, we assume 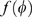 can be well-modeled by a number of piecewise cubic polynomials with boundary constraints ensuring that the entire function is smooth. Cubic splines have been previously used to regularize and simplify ill-posed integral equations [25],[26] and to represent gene expression profiles [13]. Under the cubic spline model, the expression function may be written
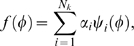
(11)where 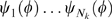 form a set of 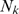 basis functions for the natural cubic splines with a particular set of knots 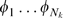. See, e.g., [27],[28], for a discussion of splines and methods of constructing the basis functions 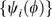. The coefficients 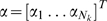 determine the particular realization of 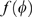 from within the family of functions spanned by the natural cubic spline basis. We choose a dense sampling of 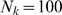 knots uniformly spread over the [0, 1] domain of 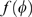. With 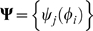 an 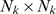 matrix, 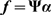 is an 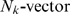 representing 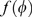 evaluated at the knot values.

In order to estimate the expression function, which is solely specified by 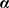 in our model, we minimize the following cost criterion
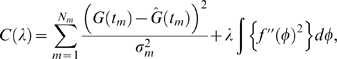
(12)where 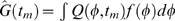. The first term is a data fidelity measure that quantifies the closeness of the model-predicted measurements to the actual measurements, weighted by the inverse of the measurement variance of each particular measurement, 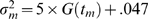 (see supplementary Text S1). The second term in Eq. (12), a second derivative cost, is a regularization term that penalizes solutions containing rapid fluctuations and is commonly used in regularizing natural smooth systems [28]–[31]. The constant 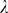 is a smoothness parameter that establishes a tradeoff between data fidelity and smoothness enforced by the second derivative norm. The smoothness parameter is chosen though cross-validation (described in the next section).

The cost function 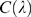 is minimized subject to two constraints

1. *Positivity constraint*. Because RNA concentrations cannot be negative, we constrain 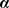 such that all the elements of 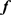 are non-negative
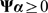
(13)
2. *Continuity constraint*. RNA concentrations must be continuous across cell division. The constraint may be concisely written as a single linear equation
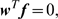
(14)where 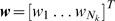 is a constraint vector that, in addition to enforcing continuity across cell division, also specifically takes into account the partitioning of mRNA according to the average relative volumes of SW and ST cells. The full development of the continuity constraint is given in the supplementary Text S1.

The final optimization problem is to minimize 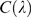 subject to the two constraints
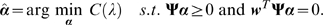
(15)As illustrated in the supplementary Text S1, the cost function 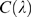 may be written as a quadratic form. For the results presented in this paper Eq. (15) was solved using the quadprog function of MATLAB's Optimization Toolbox version 4.0. The sampled estimated expression function is then given as 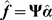, or the elements of 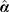 may be used in Eq. (11) to evaluate 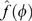 for any value of 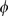.

### Cross-validation for determination of 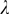


The solution to the optimization problem (Eq. (15)) depends on the value of the smoothness parameter 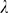: small 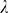 favor data fidelity and are susceptible to overfitting, large 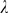 may oversmooth the estimated expression function. Cross-validation provides a principled method to select an appropriate value of 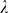. The results in this paper utilize *leave-one-out* cross-validation [28],[32] as follows.

For a fixed value of 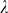, the optimization is first performed using all the data except for measurement *m* (with value 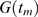). Denote the resulting estimated expression function as 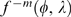. The process is repeated, excluding a different measurement each time. The total cross-validation measure
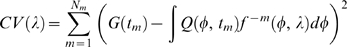
(16)is then minimized over 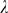 to obtain 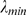, which is then used in Eq. (15) with all the data in order to obtain the optimal 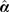 which, in turn, produces the desired expression estimate.

## Results

### Our model accurately describes the time-dependent state of a *Caulobacter* population

The cell-type distribution model enables us to mathematically determine the probability that a cell taken from a synchronized population is in a given phase. For example, the probability that a single *Caulobacter* taken from a population 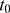 minutes following synchronization is in the SW phase is
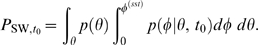
(17)However, because 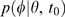 is difficult to compute directly, we may alternatively calculate various probabilities from the simulation described in the previous section.

**Figure 4:**
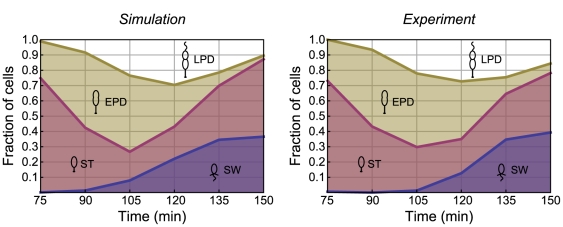
The simulated distribution of a growing, synchronized population of *Caulobacter* matches the experimentally-observed distribution. A comparison of the simulated and experimentally-determined distributions shows that the population fractions of SW cells, young ST cells, early predivisional (EPD) cells, and late predivisional (LPD) cells are similar in both. Experimental data is reproduced from Judd et al. [33].

Our simulated distribution, with cells grouped broadly into the SW, ST, EPD, and LPD types, is shown alongside the experimentally-determined distribution in Fig. 4. The ST-EPD and EPD-LPD transition phases were fixed at 0.69 and 0.87 respectively, with the mean cell-cycle time taken to be 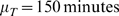 with COV = 0.13. Experimental data was reproduced from Judd et al. [33]. As can be seen in Fig. 4, our cell-type distribution model predicted highly similar fractions of SW, ST, EPD, and LPD cells. Experimentally, distinguishing between ST and EPD cells and EPD and LPD cells is difficult as the morphological differences between them are subtle, thus our assignment of those transition phases is somewhat arbitrary. The difference between SW and ST is more easily observed experimentally. Overall, our model predicted a distribution of cells that is, on average, only a few percent different from experimental observation at all time points and for all cell types.

### Extracted data show new details in essential gene expression profiles

**Figure 5:**
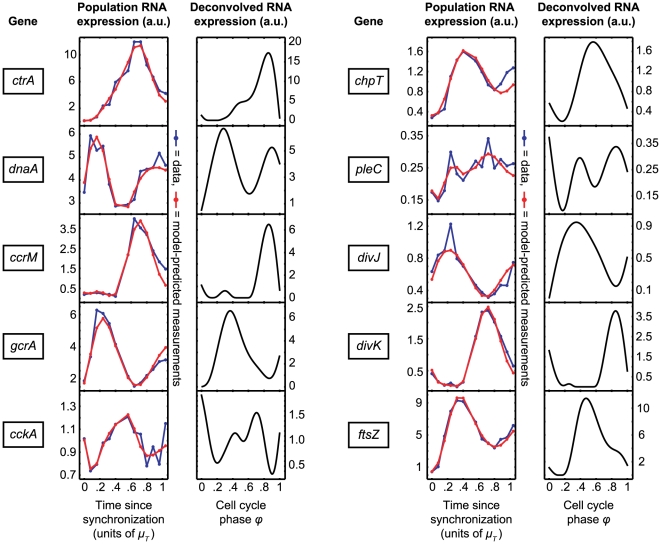
Deconvolved gene expression profiles reveal features hidden in the population-level measurements. Shown here in arbitrary units are the original microarray data (blue line), the model-predicted measurements 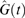 (red line), and the deconvolved profiles 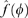 for 10 genes shown to be essential components of the *Caulobacter* cell cycle control network. Microarray data are taken from a cell-cycle Affymetrix expression data set published by McGrath et al. [38].

There are over 500 cell cycle-regulated genes in the *Caulobacter* genome [34]. In this paper we apply our deconvolution method to analyze the expressions of a subset of these: genes that are essential for cell viability or proper development and have been included in previous models of the *Caulobacter* cell cycle control network [6], [7], [35]–[37]. Microarray data for 10 cell cycle-regulated genes (*ctrA*, *dnaA*, *ccrM*, *gcrA*, *cckA*, *chpT*, *pleC*, *divJ*, *divK*, and *ftsZ*) was taken from a cell-cycle Affymetrix expression data set published by McGrath et al. [38]. The original microarray measurements, model-predicted measurements 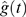, and spline-predicted profiles 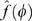 are shown in Fig. 5. The regularization parameters used, as determined by cross-validation, are listed in supplementary Table S1.

In general, the deconvolution procedure yielded expression profiles with peaks shifted to later times relative to the population data, and recovered details lost in the population averaging. For example, the deconvolved expression profile for *ctrA* remains flat until the SW-to-ST transition, and shows an expression ‘shoulder’ before the main peak around the phase of cell compartmentalization (transition from EPD-LPD). The transcription of *chpT*, *pleC*, and *ftsZ* is similarly delayed until the SW-to-ST transition. Both *ccrM* and *divK* are highly repressed until just prior to the EPD stage. Many of the genes also show a narrowing of the expression peaks. An extended analysis of these 10 deconvolved gene profiles is left for the Discussion section.

### Deconvolved gene expression profiles are robust to variability in model parameters

#### Uncertainty in mean SW-to-ST transition phase

The average 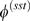 (written as 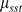) used in our model was taken from the literature, where it has been reported to be approximately 0.25 under rolled test tube conditions [17],[22] or as high as 0.33 [39],[40]. However, we are unaware of any detailed, quantitative study of the timing of SW-to-ST transition. As a major parameter in our distribution model, determination of a precise value for 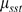 was prudent.

**Figure 6:**
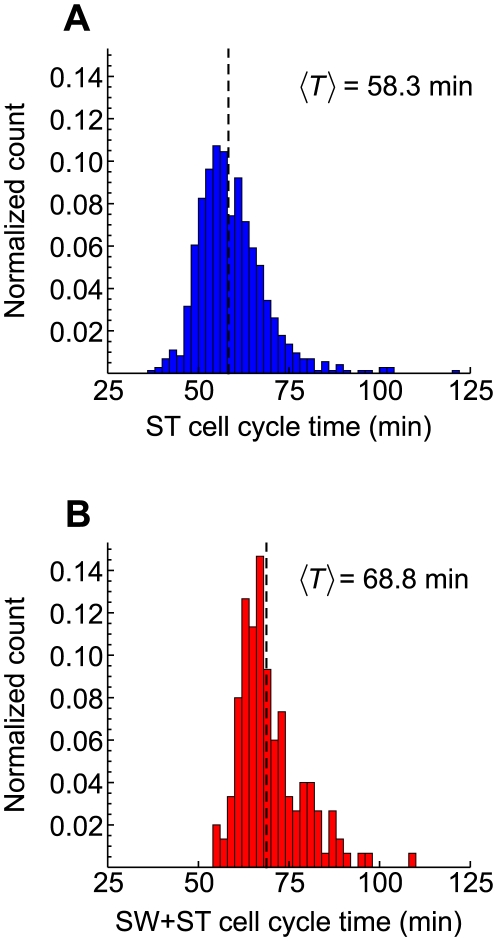
The fraction of the cell cycle spent as a SW cell is reduced considerably under rapid growth in microfluidic culture. Histograms of single-cell division times for ST cells only (A) and for the full cell cycle (B), measured under microfluidic conditions, show an average SW-to-ST transition time 10.5 minutes (difference between the two histogram means). This translates to a 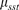 of ∼0.15 ( = 10.5/68.8), significantly less than has been previously reported.

Fortunately, the natural adhesion and asymmetric division of *Caulobacter* allow for studies of cell cycle timing in microfluidic devices with high temporal resolution [1],[41]. We used a simple microfluidic apparatus to monitor a large number of cells and determine both the full cell cycle time and the time from the SW-to-ST transition to cell division (see supplementary Text S1). This latter time period, referred to as the ST cell division time, was measured for 727 cells (Fig. 6A). The time between the first attachment of a SW and the first division of that cell, i.e., the full cell cycle time, was measured for 150 cells (Fig. 6B). The means of these two distributions are 58.3 minutes and 68.8 minutes respectively. We then arrived at an estimate of the average time the cell spends in the SW stage as 10.5 minutes, the difference between the two means. This translates to a surprising 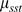 of ∼0.15 ( = 10.5/68.8), significantly lower than has been observed previously.

It is clear from our microfluidic growth assays that the mean SW-to-ST transition phase is dependent on growth and/or environmental conditions. Our choice of 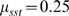 in the deconvolution of the microarray data is based on the fact that the data were taken from cells grown under standard rolled test tube conditions. However, one may not always know *a priori* the true value of 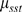 under particular environmental conditions. Thus it is worth considering what impact a mismatched 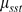 has on the estimated expression profiles.

To evaluate this impact, we replaced the 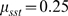 in our population distribution model with 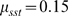 and reapplied the expression estimation routine. The various genes' expression functions calculated using 
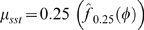
 are plotted along with the functions calculated using 
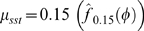
 and shown in supplementary Figure S1. Regularization parameters are listed in supplementary Table S1. The 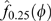 and 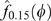 are qualitatively similar, however, to assess their quantitative differences, we discretized the functions into 100 phase points 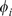 between 0 and 1 and calculated the residuals normalized by the maximum expression:
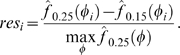
(18)We also determined the Spearman rank correlation coefficients 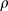 between the 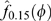 and 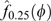. For each gene, the mean absolute value of the normalized residuals and the correlation coefficient is shown in Table 1. Despite the significant change in the SW-to-ST transition model parameter (∼40%), the average of the absolute value of the differences between 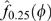 and 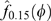 for all genes ranges from 8–12% of maximum expression. The functions are also highly correlated, with no pair exhibiting a correlation coefficient less than ∼0.77.

**Table 1:** Effect of change in model parameters on deconvolved profiles.

	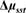	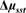	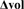	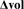
Gene name	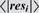	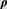	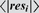	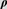
*ctrA*	0.10	0.9580	0.060	0.9846
*dnaA*	0.10	0.8882	0.022	0.9941
*ccrM*	0.11	0.8058	0.025	0.9942
*gcrA*	0.11	0.8741	0.019	0.9980
*cckA*	0.10	0.7922	0.021	0.9923
*chpT*	0.09	0.9378	0.011	0.9995
*pleC*	0.09	0.7685	0.017	0.9958
*divJ*	0.08	0.9453	0.014	0.9985
*divK*	0.10	0.8850	0.028	0.9898
*ftsZ*	0.12	0.8653	0.015	0.9986

The minimal effect of variation in model parameters is characterized by (i) the mean absolute value of the normalized residuals and (ii) the Spearman rank correlation coefficients 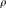 between discretized deconvolved expression functions. The change in 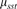 (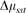) is a comparison of expression profiles 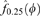 calculated with 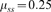 and profiles 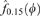 calculated with 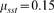. Change in cell volume (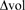) is a comparison of profiles calculated with the cell volume model 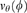 described previously (Eq. 5) with profiles calculated assuming constant cell volume.

#### Uncertainty in cell volume model

The function for the phase-dependent volume of a single cell (Eq. (5)) is an additional aspect of the model for which there has been no prior detailed investigation. We chose a reasonable piecewise linear model based on the measured average volume fraction of SW vs. ST cells, however, as with the transition phase, an analysis of the effect of changes to the single-cell volume function was warranted. We therefore reapplied the expression estimation replacing the volume function Eq. (5) with a constant cell volume, and discretized the functions into 100 phase points as before. The normalized residuals were calculated analogously to those in Eq. (18). The mean absolute value of the residuals and Spearman correlation coefficient for each gene are shown in Table 1. As can be seen in the Table, a change to a constant volume model has even less of an effect on the results of the deconvolution than the change in 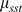. The means of the absolute values of the residuals are as low as ∼1% of maximum expression, and the functions are very highly correlated: 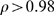 for all genes.

## Discussion

While population-level experimental techniques typically allow for high-throughput and fast data collection, they are unable to capture many of the details present at the level of single cells. This is an unavoidable consequence of population averaging; population-based data are in fact transforms of organism- and condition-specific population asynchrony kernels with single-cell data. Thus, an assumption of equivalence of population and single-cell data is an assumption of a non-physical delta function integral kernel. Recognizing this, cell distribution models have been proposed with the aim of extracting more detailed information from biological time-series data. Perhaps the simplest improvement on the delta function model is a fixed kernel such as a Gaussian. Further improvements have been made by allowing for a Gaussian kernel whose width increases with time (e.g., [13]). However, a normal distribution of this kind is not sufficient to describe the complex cell-phase distribution of organisms that undergo asymmetric division, and attempts to deconvolve single-cell expression for such organisms will lead to unreliable results. As a result, we have developed an intuitive mathematical model of the cell-type (or, alternatively, the cell-phase) distribution of asymmetrically-dividing cells as a function of time following synchronization, using *Caulobacter* as a specific example. Our model takes into account the initial population asynchrony and, similar to the yeast cell cycle phase probability density model presented in Orlando et al. [12], captures the phase variability resulting from asymmetric cell division and differences in cell cycle times. An appealing aspect of our model is its simplicity; a knowledge of three easily-measured parameters—namely the mean SW-to-ST transition phase (or equivalent), division time COV, and SW/ST cell total volume fraction (or equivalent)—and the initial synchronization state (i.e., the cell-type distribution at the outset of an experiment) are all are that is required to describe the time-dependent cell-type distribution.

The aforementioned parameters and initial synchronization state are specific to a given model system and experimental condition. For a synchronized population of *Caulobacter* under normal growth conditions, we use a mean SW-to-ST transition phase of ∼0.25, division time COV of 0.13, cell volume partitioned 40% SW to 60% ST, and a simulated initial cell cycle phase distribution that accurately models the real synchronization process. But *Caulobacter* is not the only synchronizable model system to which our cell-type distribution model can be applied. Indeed, synchronizable model systems are found across the tree of life, including *E. coli* [42], *S. cerevisiae* [43], and mammalian cells [44]. A 1957 review by Campbell describes synchronization methods for 11 microbial species [45]. For the symmetrically dividing *E. coli*, the equivalent of the SW-to-ST transition phase would be set to zero, and the two daughter cells would (on average) have the same volume. In the case of *S. cerevisiae*, the SW-to-ST transition phase equivalent is equal to the average fraction of the cell cycle that the budded daughter cell remains in the early G1 stage [46], with the average size of the budded cell being smaller than that of its mother [47]. The division time COVs for a number of commonly studied systems have already been published (a compilation of these values can be found in [1]). Initial cell distributions for many of these organisms have to be determined.

We note that we have assumed a perfect *Caulobacter* synchrony, i.e., exactly 100% of the cells at the beginning of the experiment are SW cells. In real cell synchrony experiments, SW fractions are close but not necessarily equal to 100% (see, e.g., [22]). However, minor differences in the purity of a synchronized population are not expected to significantly alter our results. That our cell-phase distribution model is consistent with experimental observations of the time-dependent state of a *Caulobacter* population (Fig. 4) supports this assumption.

Along with characterization of cell distribution, there has been considerable interest in recent years in extracting “single-cell”-like information from population data using deconvolution-type algorithms [13]–[15],[48],[49]. Although all algorithms of this kind are somewhat limited in the level of detail they can provide about biological systems—at best, only synchronous average information, and not the full stochastic variability between cells at identical phases, can be determined—they have been highly effective at uncovering features not visible in the population data. The model-based deconvolution method presented here is an extension to these previous methods and a powerful tool for the analysis of biological data, requiring no more information than the parameters described previously, and is applicable to any time-series data set for which the state of the synchrony is known or can be predicted. In particular, our method can be applied to time-series gene expression data to identify additional cell cycle-regulated genes not previously discovered and to complete meta-analyses across multiple platforms (i.e. competitive hybridization oligo arrays or non-competitive hybridization arrays such as Affymetrix). Although the differences in the data obtained from different platforms may require modifications to the kernel function, the method itself is independent of the experimental and biological details; indeed, the method supports arbitrary kernel functions.

Even with a detailed and accurate kernel and an accepted deconvolution-type algorithm, the precise shape of a deconvolved function is in general highly sensitive to the value of the regularization parameter (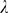 in this work; see Eq. (12)). To objectively address this problem, we employ a cross-validation routine that provides a sensible and well-established criterion for determining the appropriate amount of regularization. Our use of cross-validation in deconvolution of time-series gene expression data thus represents an improvement over methods that use arbitrary regularization based only on visual inspection of the estimated profiles.

By construction, the model-based deconvolution method presented in this paper mitigates the effects of synchronization loss in expression experiments. However, as with all time series experiments, the estimates remain dependent on the sample rate of the data. If the sample rate is insufficiently high to capture salient gene activity, important events in the expression profile may be missed. In principal, lower sampling rates may be accommodated by increasing the number of assumptions made about the expression profile to be estimated. In this paper, smoothness (Eq. (12)), positivity (Eq. (13)), and continuity (Eq. (14)) were all used to decrease the effective degrees of freedom and supply a maximal, yet realistic, amount of *a priori* information. The cubic splines support a broad class of potential expression functions, however more restrictive models could be used to supply stronger assumptions and support lower sampling rates—at the cost of potentially being overly restrictive and not capturing the true gene expression profile. See, e.g., [50] for further consideration of sample rates in temporal data.

**Figure 7:**
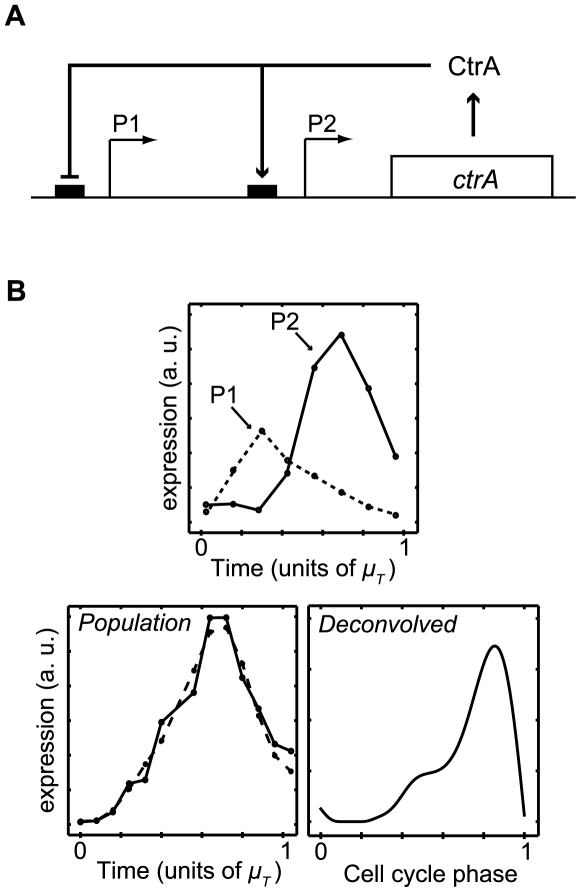
The deconvolved profile for *ctrA* reveals sequential expression from its two promoters during the cell cycle. (A) *ctrA* expression is controlled by two promoters (P1 and P2) that are differentially-regulated by the CtrA protein: the weaker P1 is negatively-controlled by CtrA and the stronger P2 is positively-controlled. (B) The (early) P1 promoter is activated immediately after replication of the *ctrA* chromosomal locus following the SW-to-ST transition. The subsequent increase in the cellular CtrA concentration activates the (late) P2 promoter, leading to an even higher concentration of CtrA and the repression of P1 (top panel, data reproduced from Reisenauer and Shapiro [53]).

The synchronous average expression profiles extracted using our generalized deconvolution algorithm are, with the effects of population asynchrony removed, a much-improved reflection of biological reality. We demonstrated this with *Caulobacter*, calculating deconvolved expression profiles for 10 genes previously found to be cell cycle-regulated and essential for cell viability or polar cell development (Fig. 5). As mentioned in Results, the deconvolved expession profiles generally have their peaks shifted to later times relative to the population data. This is to be expected, since even a perfectly-synchronized population at the outset of an experiment contains both young SW cells (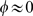) and old SW cells (and all cells in between). Many of the genes analyzed here also show a narrowing of their expression peak(s) following deconvolution, although this is not universally true. The expression profile of *divJ*, for example, is shifted to later times but not otherwise fundamentally changed; the peak, located just after the SW-to-ST transition in the deconvolved profile, is as broad as in the population measurement. Thus, expression peak narrowing is not an artifact of the deconvolution method, but rather a property of an individual gene's expression profile. Here we highlight some of our *Caulobacter*-specific results that also demonstrate the power of combining an organism-specific kernel with a generalized deconvolution routine:

***ctrA*** **.** As the master regulator of the *Caulobacter* cell cycle [51], *ctrA* is arguably the most well-characterized of *Caulobacter* genes. It has been shown that *ctrA* expression is controlled by two promoters (P1 and P2) that are differentially-regulated by phosphorylated CtrA (CtrA∼P): the weaker P1 is negatively-controlled by CtrA∼P and the stronger P2 is positively-controlled (Fig. 7A). The P1 promoter is activated in the early ST cell, immediately following replication of the chromosomal *ctrA* locus. Activation of the weak P1 promoter leads to an increase in the CtrA∼P concentration, which then activates the stronger P2 and represses P1 [52]. The differential regulation can be seen in Fig. 7B, left panel (data reproduced from Reisenauer and Shapiro [53]). Although these details are not visible in the population-level microarray data, they are revealed in the deconvolved expression profile (Fig. 7B, middle and right panels). For example, in the deconvolved profile, *ctrA* expression remains flat until DNA replication is initiated at the SW-to-ST transition. Perhaps most interestingly, the initial expression ‘shoulder’ is consistent with expression from P1, and the main peak beginning around the phase of cell compartmentalization (transition from EPDLPD), is consistent with expression from P2. The shape of the deconvolved *ctrA* profile is thus validated by our previous knowledge of the mechanism of *ctrA* regulation.

**Figure 8:**
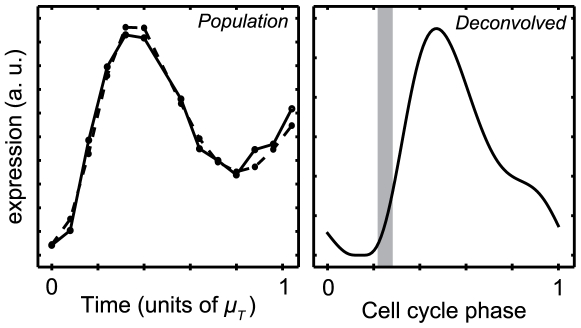
A delay in *ftsZ* expression until the SW-to-ST transition is visible in the deconvolved profile. Looking only at the population-level microarray expression data for *ftsZ*, there appears to be no delay in transcription from the time the experiment begins (left panel). However, it has been previously shown that transcription of *ftsZ* is repressed in SW cells and activated only when the DNA replication begins [20]. Repression of *ftsZ* expression in the SW phase is confirmed in the deconvolved expression profile (right panel). The gray bar indicates mean SW-to-ST transition phase +/− one standard deviation.

***ftsZ*** **.** The tubulin homolog FtsZ is essential for bacterial cell division. It has been shown that transcription of *ftsZ* is repressed in SW cells and activated only when the DNA replication begins [20]. However, this regulation is not clear from the microarray data alone. Specifically, the raw microarray data show no delay in *ftsZ* transcription from the time the experiment begins (Fig. 8, left panel). In contrast, the deconvolved expression profile reveals the delay in transcription initiation until the beginning of the ST stage (Fig. 8, right panel), consistent with our understanding of *ftsZ* regulation.

***divK*** **and** ***ccrM*** **.** DivK is an essential single-domain response regulator that is transcriptionally-activated by CtrA∼P and plays a role in the cell cycle-regulated proteolysis of CtrA [54]. The essential *ccrM* DNA methyltransferase gene [55] has an expression profile similar to that of *divK*. In both cases, deconvolution reveals that expression begins in the EPD cell, and that the change from zero to maximal expression happens over a much shorter time (i.e., the response is more switch-like) than is evident from the population data.

***cckA*** **.** One of the more interesting results is the predicted transcription profile of *cckA*, which encodes an essential histidine kinase responsible for CtrA phosphorylation [56]. The population-level microarray measurements show a single expression peak approximately half-way through the cell cycle, while the deconvolved profile shows two peaks: one beginning at the SW-to-ST transition and another peaking in the EPD cell. Although this result has not been previously reported, it does suggest the interesting possibility that *cckA* is under the control of additional and unknown layers of transcriptional regulation during the cell cycle.

These deconvolution results appear to be relatively insensitive to changes in model parameters. Of the parameters used in the cell cycle phase distribution model, the mean SW-to-ST transition phase is the one that is known with the least certainty. However, we found that precise knowledge of the mean transition phase under a given condition is not absolutely necessary for extraction of average single-cell data with our deconvolution algorithm. Even a substantial change in the assumed SW-to-ST transition phase had only a small effect on the deconvolved profiles. With respect to the single-cell volume model employed in the deconvolution algorithm, even the extreme and false assumption of fixed cell volume had an insignificant effect on the shape of the deconvolved expression profile.

One *Caulobacter*-specific result that merits further discussion is the SW-to-ST transition phase. Although accepted as around 0.25, or even up to 0.33, for standard growth in a rolling tube or shaken flask [17],[22],[39],[40], it can change under other conditions. We present data showing that the mean transition phase is reduced to 0.15 in a microfluidic environment in which the cells are rapidly growing. We recognize that a possible explanation for this low value may be that the timing of the SW-to-ST transition in our microfluidic growth experiments is skewed by a division control system in which ST cells that have just transitioned from the SW stage divide on a different time scale than ST cells that follow from cell division. However, we are not aware of any data that would suggest that this is the case. Indeed, the morphology of ST cells after the transition from SW cells appears to be the same as the morphology of ST cells after division, and a single mean SW-to-ST transition phase in our model is consistent with experimental observations (Fig. 4). Furthermore, given that a population of *Caulobacter* cells starved for carbon or nitrogen tend to arrest during the SW phase [57],[58], it is likely that the SW-to-ST transition phase can both increase and decrease, and be well above 0.33 under less-favorable environmental conditions. That the timing of this cell cycle ‘checkpoint’ may vary with growth conditions is a fascinating result that deserves more detailed study.

To our knowledge, our deconvolution method is the first to specifically deal with the unique analytical challenges posed by dimorphic organisms. Although this method can be applied to any time-series measurement made on a cellular population, we have demonstrated its utility with an analysis of cell-cycle regulated gene expression in *Caulobacter*. Certainly, directly measuring the concentration of individual transcripts in real time in single cells remains the gold standard in quantifying the gene expression behavior of single cells; the insights provided by such real-time, single-cell studies of mRNA have been profound [59]–[62]. Still, despite recent progress and a number of successes, the real-time measurement of mRNA in single cells remains a challenging problem. Our method allows for the simple analysis of mRNA concentrations measured with common laboratory tools and advances the performance of population-level methods closer to that of single-cell studies. Thus, combining high-throughput experimental expression data with novel computational algorithms can provide new and exciting insights into the function of cellular systems.

## Supporting Information

Text S1 Supporting Text
(0.08 MB PDF)

Figure S1 A comparison of expression functions calculated using *μ_sst_* = 0.25 and *μ_sst_* = 0.15 shows that they are qualitatively similar, despite the significant change in the value of *μ_sst_*. Regularization parameters are listed in Supplementary Table S1.
(0.86 MB EPS)

Video S1 The kernel structure, shown here with 0.5 minute resolution, is highly time dependent and not well-modeled by any common form.
(1.46 MB MOV)
