# Supplementary material for: Model-Based Deconvolution of Cell Cycle Time-Series Data Reveals Gene Expression Details at High Resolution
Source: PLoS Comput Biol. 2009 Aug 14;5(8):e1000460. doi: 10.1371/journal.pcbi.1000460 (PMC2718844; doi:10.1371/journal.pcbi.1000460)
Supplement: Text S1 — Supporting Text (0.08 MB PDF) [file pcbi.1000460.s001.pdf]

Model-based Deconvolution of Cell Cycle Time-series Data Reveals Gene Expression Details at High Resolution

Dan Siegal-Gaskins[1,2,3,†,*], Joshua N. Ash[4,†], Sean Crosson[5,6]
1 Mathematical Biosciences Institute, **2** Department of Plant Cellular and Molecular Biology, and **3** Plant Biotechnology Center, Ohio State University, Columbus, OH 43210 USA
4 Department of Electrical and Computer Engineering, Ohio State University, Columbus, OH 43210 USA
5 Department of Biochemistry and Molecular Biology and **6** The Committee on Microbiology, University of Chicago, Chicago, IL 60637 USA
† These authors contributed equally to this manuscript.
∗ E-mail: dsg@mbi.osu.edu

Supplementary Text S1

Quadratic form of the cost function $C(\boldsymbol{\lambda})$

The predicted value $\widehat{G}(t_m)$ of the mth measurement $G(t_m)$ may be approximated by an inner product of sampled functions

$$\widehat{G}(t_m) = \int Q(\phi, t_m) f(\phi) d\phi \approx \delta\, \boldsymbol{q}^T(t_m)\boldsymbol{f} = \delta\, \boldsymbol{q}^T(t_m)\boldsymbol{\Psi\alpha} \ , \tag{S1}$$

where $\boldsymbol{q}(t_m) = [Q(\phi_1, t_m) \ldots Q(\phi_{N_k}, t_m)]^T$ and $\delta = 1/N_k$ is the knot spacing.

The cost function $C(\lambda)$, given in Models as:

$$C(\lambda) = \sum_{m=1}^{N_m} \frac{(G(t_m) - \widehat{G}(t_m))^2}{\sigma_m^2} + \lambda \int \{f''(\phi)^2\} d\phi \ , \tag{S2}$$

with $\widehat{G}(t_m) = \int Q(\phi, t_m) f(\phi) d\phi$, may be written as a quadratic form in $\boldsymbol{\alpha}$ as follows: define $\boldsymbol{Q}$ as the $N_m \times N_k$ measurement matrix $\boldsymbol{Q} = [\boldsymbol{q}(t_1) \ldots \boldsymbol{q}(t_{N_m})]^T$ that maps the sampled expression function $\boldsymbol{f}$ to the predicted measurements $\widehat{\boldsymbol{g}} = [\widehat{G}(t_1) \ldots \widehat{g}(t_{N_m})]^T$

$$\widehat{\boldsymbol{g}} = \boldsymbol{Qf} = \boldsymbol{Q\Psi\alpha} \ . \tag{S3}$$

In Eq. (S3) and what follows, we absorb the height scaling constant δ into the expression function f that is to be estimated. With $f(\phi) = \sum_{i=1}^{N_k} \alpha_i \psi_i(\phi)$, the second term of Eq. (S2) may be written

$$\lambda \boldsymbol{\alpha}^T \boldsymbol{\Omega\alpha} \ , \tag{S4}$$

where $\boldsymbol{\Omega} = \{\Omega_{ij}\}, \Omega_{ij} = \int \psi_i''(\phi)\psi_j''(\phi) d\phi$. Letting $\boldsymbol{R}$ be a diagonal matrix of measurement variances $\sigma_1^2 \ldots \sigma_{N_m}^2$, the cost function (Eq. (S2)) may be written

$$C(\lambda) = (\boldsymbol{g} - \boldsymbol{Q\Psi\alpha})^T \boldsymbol{R}^{-1} (\boldsymbol{g} - \boldsymbol{Q\Psi\alpha}) + \lambda \boldsymbol{\alpha}^T \boldsymbol{\Omega\alpha} \ , \tag{S5}$$

which is a quadratic form in $\boldsymbol{\alpha}$.

Justification for use of the synchronous average expression function $\boldsymbol{f}(\boldsymbol{\phi})$

It has been shown that as a result of noise in gene expression, the levels of expression (measured with fluorescent reporters) are normally-distributed about the population mean [1]. Letting $f(\phi) =$

$(1/N)\sum_k f_k(\phi)$ denote the average population expression, we may then write the single-cell expression function $f_k(\phi)$ for cell k as the product of a scaling factor s_k with the population average

$$f_k(\phi) = s_k\, f(\phi), \tag{S6}$$

where s_k is independent of all other cell parameters θ, ϕ and is drawn from a normal distribution with mean 1. For a given species of RNA, the total number of transcripts in the population at time t is then given by:

$$\begin{aligned} R(t) &= \sum_{k=1}^{N(t)} f_k(\phi) v_k(\phi) \\ &= \sum_{k=1}^{N(t)} (s_k \times f(\phi)) v_k(\phi) \\ &\approx N(t) E_{\theta,\phi,s}[s f(\phi) v_\theta(\phi)] \\ &= N(t) E_s[s] E_{\theta,\phi}[f(\phi) v_\theta(\phi)] \\ &= N(t) \int f(\phi) \tilde{Q}(\phi, t)\, d\phi \ , \end{aligned} \tag{S7}$$

where $E_X[\ldots]$ denotes statistical expectation over the random variable X. As s is random and independent of θ and ϕ, the expectation of the product of s and $f(\phi)v_\theta(\phi)$ is equal to the product of their expected values (1 and $\int f(\phi)\tilde{Q}(\phi, t)d\phi$, respectively).

Microarray data noise model

It has been previously shown that the dominant source of noise in experiments using Affymetrix microarrays is hybridization noise [2]. This leads to a signal variance on each gene expression value that is, above a certain threshold, proportional to the value itself,

$$\sigma^2_{G>G_0} \simeq \beta \times G \ , \tag{S8}$$

where G is the level of gene expression, G_0 is the threshold, and β is a constant. Although the experiments in [2] use mRNA from a human Burkitts lymphoma cell line, it was suggested that the hybridization noise component proportional to signal intensity does not depend on the type of genechip and the sample being used. Consistent with that work, we used a noise model with β=5. Also, the threshold is the 10th percentile of all expression values, which, for the entire *Caulobacter* data set analyzed here, is 0.188. Far below this threshold, i.e., at low levels of expression, we assumed that the variance is constant. To combine these two limits into a single noise model, we assumed that at the threshold intensity the variance diverges from proportionality by 5%, so that

$$\begin{aligned} \sigma^2 &= 5 \times G + 0.05 \times (5 \times 0.188) \\ &= 5 \times G + .047 \ . \end{aligned} \tag{S9}$$

Constraints on the average single-cell expression functions

There are typically multiple solutions to inversion problems of the kind used here. We therefore constrained the single-cell expression profiles by excluding non-physical solutions for which RNA concentration became negative. We also applied a continuity constraint, since for every cell k the concentration of

any RNA species at $\phi = 1$ must be equal to the volume-weighted sum of concentrations at $\phi = 0$ and $\phi = \phi_k^{(sst)}$. Mathematically, we have that

$$\begin{aligned} f_k(1) &= \frac{v_k(0)f_k(0) + v_k(\phi_k^{(sst)})f_k(\phi_k^{(sst)})}{v_k(0) + v_k(\phi_k^{(sst)})} \\ &= 0.4f_k(0) + 0.6f_k(\phi_k^{(sst)}) \ , \end{aligned} \tag{S10}$$

where $f_k(\phi)$ and $v_k(\phi)$ are the expression in and volume of cell k at phase ϕ. We use the previously established values of 0.4 and 0.6 for the average SW and ST cell volume fractions [3]. The synchronous average expression over N cells is $f(\phi) = (1/N)\sum_k f_k(\phi)$, which we apply to the left- and right-hand sides of Eq. (S10) to get

$$f(1) = 0.4f(0) + 0.6\frac{1}{N}\sum_k f_k(\phi_k^{(sst)}) \ . \tag{S11}$$

As before, we assume that the variability between the f_k is independent of $\phi^{(sst)}$, and thus we replace the individual f_k with their mean value (see Supplementary Information), and rewrite Eq. (S11) as

$$\begin{aligned} 0 &\approx 0.4f(0) + 0.6\frac{1}{N}\sum_k f(\phi_k^{(sst)}) - f(1) \\ &\approx 0.4f(0) + 0.6\int f(\phi^{(sst)})p(\phi^{(sst)})d\phi^{(sst)} - f(1) \\ &= 0.4f(0) + 0.6\int f(\phi^{(sst)})\mathcal{N}(\phi^{(sst)};\mu_{sst},\sigma_{sst}^2)d\phi^{(sst)} - f(1) \\ &\approx 0.4f(\phi_1) + 0.6/N_k\sum_{i=1}^{N_k} f(\phi_i)\mathcal{N}(\phi_i;\mu_{sst},\sigma_{sst}^2) - f(\phi_{N_k}) \\ &= \boldsymbol{w}^T\boldsymbol{f} \ , \end{aligned} \tag{S12}$$

where $\mathcal{N}(\phi^{(sst)};\mu_{sst},\sigma_{sst}^2)$ is a Gaussian probability density function evaluated at $\phi^{(sst)}$, $\phi_1 = 0$, $\phi_{N_k} = 1$, and $\boldsymbol{w} = [w_1 \ldots w_{N_k}]^T$, with

$$w_i = \begin{cases} 0.4 + (0.6/N_k)\ \mathcal{N}(0;\mu_{sst},\sigma_{sst}^2), & i = 1 \\ (0.6/N_k)\ \mathcal{N}(\phi_i;\mu_{sst},\sigma_{sst}^2), & 1 < i < N_k \\ (0.6/N_k)\ \mathcal{N}(1;\mu_{sst},\sigma_{sst}^2) - 1, & i = N_k \end{cases} . \tag{S13}$$

Division time assays

To determine the division times for *Caulobacter* SW and ST cells, we used a simple microfluidic apparatus and followed the microfluidic protocol described previously [4,5]. ST cells attach to the glass surface of the microfliuidic via the adhesive holdfast and are oriented along the direction of the flow of growth medium. Following cell division, ST cells remain attached to the surface while the majority of daughter SW cells are flushed out of the channel. ST cells continue to grow and divide for the duration of the experiment. The time between division events is then the ST cell division time. Following division events, SW cells do occasionally attach to the glass cover slip prior to separation, and remain attached downstream of the ST cells when division is complete. The cells then stay attached to the surface as they transition from the SW to the ST phase, and the stalk and holdfast develop. The time between the first attachment of a SW and the first division of that cell is then the full cell cycle time.

Cell division took place in microfluidic channels measuring 200 μm wide by 50 μm deep by 2 cm long and made with polydimethylsiloxane (PDMS, Sylgard Brand 184 Silicone Elastomer Kit). The PDMS

and glass cover slip were cleaned and sealed using a Plasma Prep II plasma cleaner (SPI Supplies). Sodium hydroxide (2M solution), ethanol, and water were sequentially flowed into the channels to clean the interior before cell loading.

Individual colonies of wild-type *Caulobacter crescentus* strain CB15 [6] were taken from a peptone/yeast extract (PYE)-agar plate and grown overnight in 5 ml PYE medium at 30°C, diluted to 0.1 optical density at 660 nm (OD_{660}) and regrown for 2 hours. Cells were then loaded into the microfluidic chamber and incubated for an additional hour prior to imaging. A Harvard Apparatus PHD2000 infuser was used to induce a constant flow of PYE medium at a rate of 12 μl/min for the duration of the experiment.

Cells were imaged with a Leica DM5000 at 630x magnification in phase contrast mode. Images were collected at 2 minute intervals on a Hamamatsu Orca-ER digital camera, and the light dosage was limited to 200 msec exposure and ∼5 second manual focus time per exposure. The temperature in the room was maintained at 30°C. Cell growth and division were monitored for 12-14 hours during each of four independent experimental runs.

Images were imported into ImageJ (National Institutes of Health, Bethesda, MD) for processing. The images were converted into binary stacks by subtracting the image backgrounds and adjusting the threshold pixel intensity. Cell areas were calculated in ImageJ, and data were further analyzed using Mathematica (Wolfram Research, Inc., Champaign, IL).

References

1. Ozbudak EM, Thattai M, Kurtser I, Grossman AD, van Oudenaarden A (2002) Regulation of noise in the expression of a single gene. Nat Genet 31: 69 - 73.

2. Tu Y, Stolovitzky G, Klein U (2002) Quantitative noise analysis for gene expression microarray experiments. Proc Natl Acad Sci USA 99: 14031-14036.

3. Thanbichler M, Shapiro L (2006) MipZ, a spatial regulator coordinating chromosome segregation with cell division in caulobacter. Cell 126: 147–162.

4. Siegal-Gaskins D, Crosson S (2008) Tightly-Regulated and Heritable Division Control in Single Bacterial Cells. Biophys J 95: 2063 - 2072.

5. Purcell EB, Siegal-Gaskins D, Rawling DC, Fiebig A, Crosson S (2007) A photosensory two-component system regulates bacterial cell attachment. Proc Natl Acad Sci USA 104: 18241 - 18246.

6. Nierman WC, Feldblyum TV, Laub MT, Paulsen IT, Nelson KE, et al. (2001) Complete genome sequence of *Caulobacter crescentus*. Proc Natl Acad Sci USA 98: 4136-4141.

Supplementary Table S1

Gene name	$\lambda_{0.25}$	$\lambda_{0.15}$
ctrA	2.73×10^{-7}	2.89×10^{-6}
dnaA	7.02×10^{-7}	1.80×10^{-6}
ccrM	1.13×10^{-6}	7.44×10^{-6}
gcrA	7.02×10^{-7}	4.64×10^{-6}
cckA	1.06×10^{-7}	1.80×10^{-6}
chpT	3.07×10^{-5}	7.90×10^{-5}
pleC	1.19×10^{-5}	4.92×10^{-5}
divJ	1.91×10^{-5}	1.19×10^{-5}
divK	2.89×10^{-6}	1.19×10^{-5}
ftsZ	1.19×10^{-6}	1.80×10^{-6}

Regularization parameters determined by cross validation for $\mu_{sst} = 0.25$ ($\lambda_{0.25}$) and $\mu_{sst} = 0.15$ ($\lambda_{0.15}$).
